# Increased Breadth and Depth of Cytotoxic T Lymphocytes Responses against HIV-1-B Nef by Inclusion of Epitope Variant Sequences

**DOI:** 10.1371/journal.pone.0017969

**Published:** 2011-03-28

**Authors:** Morgane Rolland, Nicole Frahm, David C. Nickle, Nebojsa Jojic, Wenjie Deng, Todd M. Allen, Christian Brander, David E. Heckerman, James I. Mullins

**Affiliations:** 1 Department of Microbiology, University of Washington, Seattle, Washington, United States of America; 2 Massachussets General Hospital, Harvard Medical School, Boston, Massachusetts, United States of America; 3 AIDS Research Institute IrsiCaixa - HIVACAT, Hospital Germans Trias i Pujol, Badalona, Barcelona, Spain; 4 Institució Catalana de Recerca i Estudis Avançats (ICREA), Barcelona, Spain; 5 Microsoft Research, Redmond, Washington, United States of America; Dana-Farber Cancer Institute, United States of America

## Abstract

Different vaccine approaches cope with HIV-1 diversity, ranging from centralized^1–4^ to variability-encompassing^5–7^ antigens. For all these strategies, a concern remains: how does HIV-1 diversity impact epitope recognition by the immune system? We studied the relationship between HIV-1 diversity and CD8^+^ T Lymphocytes (CTL) targeting of HIV-1 subtype B Nef using 944 peptides (10-mers overlapping by nine amino acids (AA)) that corresponded to consensus peptides and their most common variants in the HIV-1-B virus population. IFN-γ ELISpot assays were performed using freshly isolated PBMC from 26 HIV-1-infected persons. Three hundred and fifty peptides elicited a response in at least one individual. Individuals targeted a median of 7 discrete regions. Overall, 33% of responses were directed against viral variants but not elicited against consensus-based test peptides. However, there was no significant relationship between the frequency of a 10-mer in the viral population and either its frequency of recognition (Spearman's correlation coefficient ρ = 0.24) or the magnitude of the responses (ρ = 0.16). We found that peptides with a single mutation compared to the consensus were likely to be recognized (especially if the change was conservative) and to elicit responses of similar magnitude as the consensus peptide. Our results indicate that cross-reactivity between rare and frequent variants is likely to play a role in the expansion of CTL responses, and that maximizing antigenic diversity in a vaccine may increase the breadth and depth of CTL responses. However, since there are few obvious preferred pathways to virologic escape, the diversity that may be required to block all potential escape pathways may be too large for a realistic vaccine to accommodate. Furthermore, since peptides were not recognized based on their frequency in the population, it remains unclear by which mechanisms variability-inclusive antigens (i.e., constructs enriched with frequent variants) expand CTL recognition.

## Introduction

Despite setbacks in the development of CTL-based HIV vaccines [1], it seems likely that this approach will remain a crucial component of future vaccine strategies given amassed evidence on the critical role of the cell-mediated host immune response against HIV-1 [2], [3], [4], [5], [6]. Certain characteristics of CTL responses attest to their importance, e.g., the emergence of CD8+ T lymphocytes has been temporally linked with the decline in viremia in early infection [2], [4]; some MHC class I alleles are associated with HIV-1 disease outcomes [7], [8], [9]; CTL targeting of specific proteins/epitopes, such as within Gag, has been associated with control of viral load [10], [11]. While it is apparent that antiviral CTL immune responses vary in their ability to contain HIV-1 replication, there is still no precise definition of mechanisms of protection against HIV-1 disease progression and, as such, no rational path toward a successful CTL-based vaccine.

Post-Step trial suggestions emphasize the potential benefit of strategies that would improve T cell breadth [1]. Such vaccine strategies have been proposed, e.g., for HIV-1 the mosaic [12] and COT+ [13] approaches, or the epitome for Hepatitis C [14]. These vaccine designs compress HIV-1 diversity to maximize the coverage of circulating strains: the proposed inserts include the most common variants of HIV-1, thereby infecting strains will be more likely to match or be genetically closer to the vaccine antigen. The rationale is that the efficacy of variability-inclusive vaccine will be maximized because peptides identical between the antigen and the breakthrough strain would be recognized by T cells primed by the vaccine upon breakthrough infection. Indeed, two recent studies demonstrated that mosaic HIV-1 vaccines increased the breadth and depth of cellular immune responses in Rhesus monkeys [15], [16], especially in the case of bivalent mosaic HIV-1-B Gag, Pol and Env antigens expressed by recombinant adenovirus serotype 26 vectors [15].

Although it is agreed that HIV-1's extensive variability is a major challenge for a successful vaccine strategy, which must control extremely diverse viral strains, the impact of HIV-1 diversity on CTL targeting remains poorly understood. Our knowledge of HIV-1 diversity and CTL recognition is mostly derived from whole genome mapping studies of ELISpot responses using consensus peptides and small studies of specific epitopes in HIV-1-infected individuals with longitudinal follow-up, particularly in the context of CTL escape.

We sought to formally analyze the effect of HIV-1 diversity on CTL targeting of Nef, as it comprises both conserved and variable segments and is often targeted both during acute/early and chronic infection: several Nef peptides were recognized by more than 40% of individuals in large cohorts of Americans or South-Africans infected with HIV-1 subtype B and C, respectively [11], [17]. Based on accumulated sequence data from the Los Alamos HIV Database (HIVDB) for HIV-1 subtype B Nef, we synthesized peptides corresponding to the consensus sequence and several naturally occurring mutants. The peptide set also included all peptides derived from a 3-gene-length Nef COT+ antigen [13]: with the COT+ strategy, the initial gene-length corresponds to a Center-Of-Tree (COT) sequence [18] then common 9-mer Nef variants are appended based on their frequency among circulating viruses until the pre-set length of the construct is reached. In total, the peptide library contained 944 Nef peptides which were tested for immune recognition in HIV-1 infected individuals in order to assess the impact of HIV-1 diversity on CTL recognition and to assess the improvement in viral epitope recognition afforded by a theoretical variability-inclusive vaccine candidate.

Here, we identified novel HIV-1 Nef epitopes and showed that there is no general relationship between the frequency of an HIV-1 peptide among circulating sequences and its frequency of recognition in the cohort. These findings challenge immunogen designs that are based on a “frequency-only” criterion for variant inclusion and warrant further studies into the determinants of CTL variant recognition in HIV-1.

## Materials and Methods

### Study Subjects

Subjects with chronic HIV infection were recruited at three hospitals in the Boston area. All human subject protocols were approved by the Partners Human Research Committee, and all subjects provided written informed consent prior to enrollment.

### HIV-1 subtype B Nef Peptides

Nine hundred and forty four 10-mer peptides overlapping by 9 AA were synthesized and used in the present study. These sequences cover the distribution of HIV-1 B viral sequences based on a previously described [13] dataset of 169 sequences available in the HIVDB. These variants included: i) peptides corresponding to the full-length consensus subtype B 2004 Nef; ii) peptides corresponding to the full-length COT subtype B [18]; iii) 10-mers corresponding to three natural HIV-1 B sequences optimized for combined sequence coverage (‘3-Best’) (GenBank ids: U34603, AF004394, DQ121883), and iv) 10-mers covering the 3-gene-length Nef COT+ HIV-1 B antigen (the first gene length corresponds to COT, two additional gene lengths correspond to high-frequency peptides; COT+ peptides also include five artificial 10-mers that are a sequel of the COT+ design strategy) [13]. We chose 10-AA-long peptides to increase sensitivity for *in vitro* IFN-γ ELISpot assays and to better discriminate responses to multiple partly overlapping peptides from individual discrete specificities.

### Variability metrics

Shannon entropy. Shannon entropy was used to score the variability at each position of an alignment of HIV-1 circulating sequences [19]. Five hundred and fourteen independent Nef HIV-1 subtype B sequences were gathered from the HIVDB. Shannon entropy values were determined at each site in the AA-alignment and average Shannon entropy values were calculated for each peptide over the corresponding positions.

Population frequency. We derived all unique 10-mers in the 514-sequence-dataset and defined their population frequency, i.e., the percentage of sequences with the precise 10-mer sequence present in the dataset of 514 Nef sequences.

### IFN-γ ELISpot assays

IFN-γ ELISpot assays were performed on freshly isolated peripheral blood mononuclear cells (PBMC) from 26 individuals. All peptides were tested in separate wells of the Elispot plates. Due to the large number of peptides tested, not all blood draws yielded sufficient PBMC to test at 100,000 cells/well, therefore assays were run with 74,000 to 100,000 cells/well (median 100,000 cells/well). To be scored as positive, a response had to be greater than: a) four times the mean background, b) the mean background plus three standard deviations, c) five spots per well and d) 55 spot-forming cells per million (SFC/M).

### Cross-reactivity model

The HIV-1-B Nef peptides that were reactive in the ELISpot assays were compared by testing k-mers (with k = 8, 9, 10 AA) that had the same HIV-1 HXB2 strain coordinates; in brief, a consensus k-mer was compared to its variant k-mer(s), with k being of the same length for the consensus and variant to avoid the creation of gaps when aligning the 2 k-mers. Pairs of peptides were tested when the database frequency of one of the peptides was at least ten-fold greater than that of the other k-mer. For each given 10-mer peptide pair, cross-reactivity was also assessed for the two pairs of 9-mers and three pairs of 8-mers nested in the 10-mer. We computed a cross-reactivity fraction corresponding to the number of individuals who recognized the less frequent peptide divided by the number of individuals who recognized the more common one. AA substitutions were characterized as being conservative, semi-conservative and non-conservative based on the Dayhoff PAM250 matrix [20].

## Results

### Design of 944 peptides in Nef HIV-1 Subtype B

Most Nef peptides are unique or very rare at the population level. We dissected 514 Nef HIV-1 B sequences into overlapping 10-mers and found 19,860 unique 10-mers: a small fraction of relatively frequent peptides and a long tail of rare 10-mers. More than 2/3 of all of these peptides were engendered by private mutations, i.e., 13,574 peptides were found only once in this dataset (Figure S1). For the experimental assays, 944 10-mers overlapping by 9 AA had been designed to cover a fraction of the diversity of HIV-1 subtype B Nef sequences in the population, based on a previously described dataset of 169 sequences [13]. Five synthetic peptides were integrated in the set that were unnatural junctional peptide sequels of the COT+ design, however, they were not recognized in our cohort. When all peptides were mapped along the Nef protein based on their corresponding start positions in the HXB2 reference sequence, consensus peptides covered the full length of Nef and a median of four additional peptides represented the variants found for each consensus peptide, with up to eight variants for the three most diverse segments of Nef (Figure S2).

### Identification of novel HIV-1 Subtype B Nef epitopes

The 944 Nef peptides were tested by IFN-γ ELISpot assays using fresh PBMC from 26 HIV-1 infected persons (Table S1). Viral loads did not influence the recognition by IFN-γ ELISpot assays: among untreated subjects, there was no significant relationship between viral load and either the number of ELISpot responses (r^2^ = 0.05; p = 0.001) or the number of epitopes recognized per individual (r^2^ = 0.04; p = 0.001).

**Figure 1:**
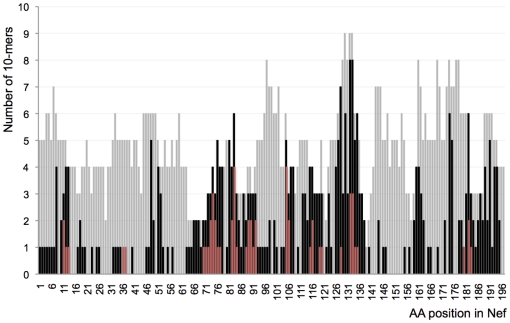
Distribution of reactive 10-mers and known epitopes in Nef. The number of 10-mers that were tested are represented as gray bars. Black bars correspond to the 10-mers that were recognized at least by one individual in our cohort. Red bars represent known epitopes that had previously been reported in the HIVDB. Each bar is placed at the start position of each 10-mer based on their HXB2 coordinates.

While 944 peptides were tested by IFN-γ ELISpot assays, only 350 elicited a response in at least 1 individual (Figure 1). Considering the 38 known epitopes reported in the HIVDB (as of August 31, 2009), 34 epitopes were recognized in our cohort at least once and not necessarily in the context of the originally described HLA allele (for 10 epitopes, recognition occurred exclusively through alleles different from the originally described ones). Considering that three of the not-targeted epitopes were 11-mers whose recognition could not be effectively tested with our 10-mer peptide library, the only previously described epitope that was not recognized in our cohort was the B*4001/B50-restricted epitope LEKHGAITS (Nef 37–45), although 3 individuals presented the HLA allele B40 (only 2-digit HLA data was available for individuals in our study).

On an individual peptide basis, the median number of ELISpot responses per subject was 21 (interquartile range (IQR) 14–41; range 1–123). Due to the multiplicity of peptide variants tested, several responses can be scored for the same 10-AA segment. Additionally, due to the 1AA-offset between the peptides, the same epitope can be found in a suite of immediately adjacent overlapping 10-mers: for example, an 8-AA-long epitope is present in three consecutive 10-mers. In order to count only the ELISpot responses that were reflective of an independent CTL specificity, we scored only one response per 10-AA segment, and, if we found two or three responses to consecutive 10-mers, we scored them as one independent response (four consecutive responses were counted as two independent responses). Hence, the median number of independent epitopes recognized per individual was seven (IQR 4–13; range 1–32).

### Benefit of a coverage-optimized peptide set

To assess whether using coverage-optimized HIV-1 subtype B vaccine inserts would engender broader and deeper CTL responses in individuals, peptides covering three potential vaccine insert strategies were compared: COT+, ‘3-Best’ and consensus. A three-gene length COT+ corresponded to 561 peptides in our test set. The ‘3-Best’ natural HIV-1 Nef strains, which were selected from the HIVDB to afford the highest coverage of HIV-1 variability [13], corresponded to 522 peptides. The Nef HIV-1 subtype B 2004 consensus and COT sequences corresponded to 197 peptides.

When we evaluated the number of CTL responses corresponding to the 3 different Nef vaccine strategies in our cohort, we found that more peptides matching the 3-gene-length COT+ were targeted than peptides contained in the other strategies: n_COT+_ = 221, n_‘3-best’_ = 200, n_CON04_ = 95, n_COT_ = 96. However, the percentage of COT+ or ‘3-best’ peptides recognized was similar (39 and 38%, respectively) while the fraction of HIV-1-B 2004 consensus or COT peptides recognized was, as expected, higher −48 and 49% of the set, respectively. Thus, if a single protein length peptide set is used, consensus or COT peptides are optimal for detecting responses, yet the breadth of T cell responses is extended with the use of coverage-optimized peptides.

### Increased ELISpot recognition using epitope variants albeit with diminishing returns

**Figure 2:**
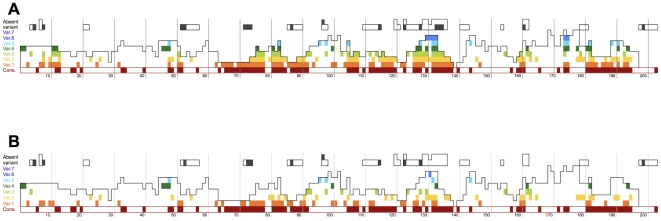
Distribution of reactive consensus and variant 10-mer peptides. Colored blocks correspond to the 10-mers that were recognized, while the 10-mers that were tested are outlined in black. Recognition of peptides is figured using a gradient of colors: consensus peptides are figured in burgundy and variant peptides follow a gradient from the most conserved (in orange) to the most rare (in purple), while peptides not found in a database of 514 sequences are in black. Each block represents the start position of the 10-mers based on their HXB2 coordinates. Panel A shows all the peptides recognized, panel B represents for each individual only the most frequent peptide recognized at each position (i.e., if an individual recognized the consensus peptide and variants 1 and 4, only the recognition through the consensus is figured).

We analyzed how each 10-mer segment of HXB2 was recognized to evaluate whether recognition was due to the consensus peptide, one or more variant peptide(s) or a combination of consensus and variants. We rank-ordered peptides based on their frequency in the database calling ‘variant 1’ the peptides corresponding to the most common epitopic variant (after the consensus) found in circulating sequences, ‘variant 2’ corresponds to the second most frequent peptides, and so on. Figure 2 represents whether and how each of the 203 10-AA stretches covering HXB2 was recognized. One hundred and thirty seven segments were recognized, while 66 segments were not recognized in our cohort despite the use of multiple variants. Figure 2A highlights two highly immunogenic regions (centered around positions 80 and 130) that elicited responses to both consensus and variant peptides. Consensus peptides were more often recognized than variant peptides: 91 of 203 consensus peptides or 45% were recognized (Table 1). The percentage of recognition dropped with the frequency of the variants in the circulating population: to 41% for ‘variant 1’ peptides and to 26% for ‘variant 4’ peptides. The recognition of ‘variants 5, 6, 7’ or ‘variants absent in the database’ (i.e., none of the 514 sequences in the dataset included these variants) did not appear to correlate with their frequency in the population: none of the variant 7 peptides were recognized, while 16 of the 43 (37%) variant peptides absent in circulating sequences were recognized.

**Table 1:** ELISpot recognition of consensus and variant peptides.

Counting all responses									
	Cons.	Var. 1	Var. 2	Var. 3	Var. 4	Var. 5	Var. 6	Var. 7	Absent
Peptides recognized	91	81	65	40	24	14	6	0	16
Peptides not recognized	112	115	110	99	68	28	10	4	43
Peptides tested	203	196	175	139	92	42	16	4	59
% peptides recognized	44.83	41.33	37.14	28.78	26.09	33.33	37.50	0.00	27.12

Counting responses to the consensus and additional responses afforded by the addition of variants									
	Cons.	Var. 1	Var. 2	Var. 3	Var. 4	Var. 5	Var. 6	Var. 7	Absent
Peptides recognized	91	50	33	12	10	5	1	0	10
Peptides not recognized	112	146	142	127	82	37	15	4	49
Peptides tested	203	196	175	139	92	42	16	4	59
% peptides recognized	44.83	25.51	18.86	8.63	10.87	11.90	6.25	0.00	16.95

To better characterize the coverage enhancement afforded by inclusion of variant peptides, we scored responses to a 10-mer only once for each individual, i.e., if an individual responded to the consensus we did not count responses to the other variants tested. Figure 2B reveals the sparsity of the reactivity afforded by some variants, underlining that variant peptides do not elicit the same degree of recognition in the population as consensus peptides. Table 1 shows that although 41% of variant 1 peptides were recognized, only 25% of these peptides elicited responses in individuals who did not recognize the consensus. With the decrease in frequency of the variants, there was a diminishing number of new responses observed. Twenty-nine percent of variants 3 and 26% of variant 4 peptides were recognized, allowing the detection of responses in 9 and 11% more individuals, respectively (who had not recognized the consensus or more frequent variants).

**Figure 3:**
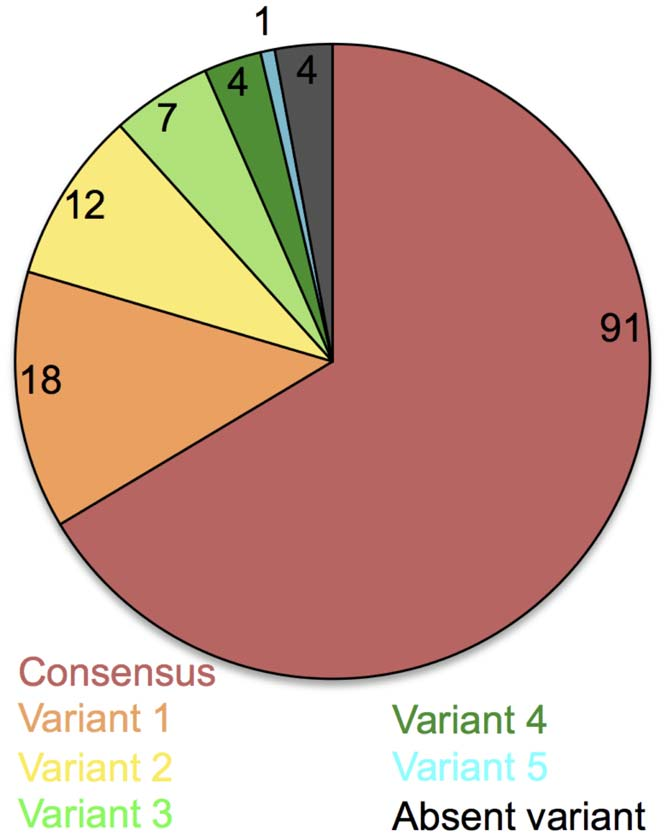
Distribution of responses between consensus and variant 10-mers. The pie chart represents the 137 HIV-1 segments that were recognized in our cohort and the proportion of these that were recognized through the consensus 10-mers or through any of the variant peptides.

In summary, each additional variant level tested yielded smaller increases in the overall spectrum of recognition. Figure 3 shows the proportion of responses to the 203 segments (n = 137) that were identified by recognition of consensus (n = 91) or variant (n = 46) peptides. Of the variant peptide responses, 18 were elicited by ‘variants 1’, 12 more responses were detected using the second most common variants, while identification of the last 16 responses required seven more levels of variation.

### Similar magnitudes of ELISpot responses against consensus or variant peptides

We compared the average magnitude of responses toward the consensus and variant peptides. Ninety-one consensus peptides were recognized with an average magnitude of 292 SFC/M (median = 180 SFC/M), while 246 variant peptides were recognized with an average magnitude of 243 SFC/M (median = 170 SFC/M). Hence, the consensus and variant epitopes elicited responses of similar magnitudes (p = 0.15) (Figure S3).

**Figure 4:**
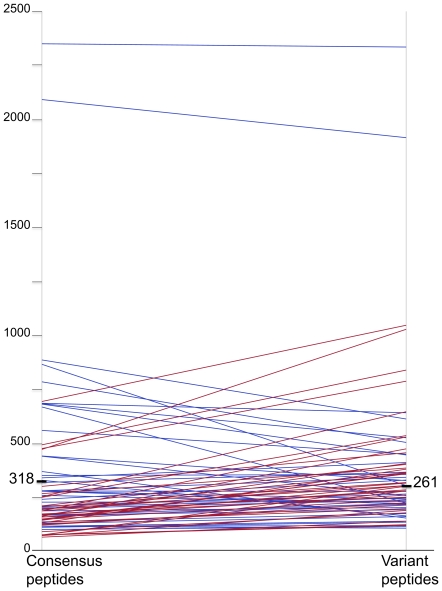
Magnitude of IFN-γ ELISpot responses toward consensus and variant for paired sets of peptides. Responses of decreasing magnitude between the consensus and variant peptide are figured in blue, responses of increasing magnitude are figured in red.

Next, we analyzed the magnitude of responses for pairs of consensus plus variant peptides, i.e., the average magnitude found for the consensus peptide was compared to the average magnitude elicited by the different variant peptides beginning at the same position (irrespective of whether the individual was able to elicit responses against both consensus and variant forms). There were 73 paired sets of consensus/variant(s) that were recognized in the cohort. The average magnitude elicited by the consensus (318 SFC/M) or the variant peptides (261 SFC/M) were not significantly different: p = 0.80 (Figure 4).

Last, we analyzed the magnitude of responses on an individual basis, focusing on individuals who mounted responses toward both the consensus and one or more of the variant peptides. Of the 91 peptides that were recognized using consensus peptides, variants of 44 of these were recognized by at least one individual (and up to 11 individuals). There was no significant difference in the magnitude of the responses elicited for the consensus or for a variant (p = 0.70).

### Peptides that matched the subjects' HIV-1 sequences elicited responses of higher magnitude

**Figure 5:**
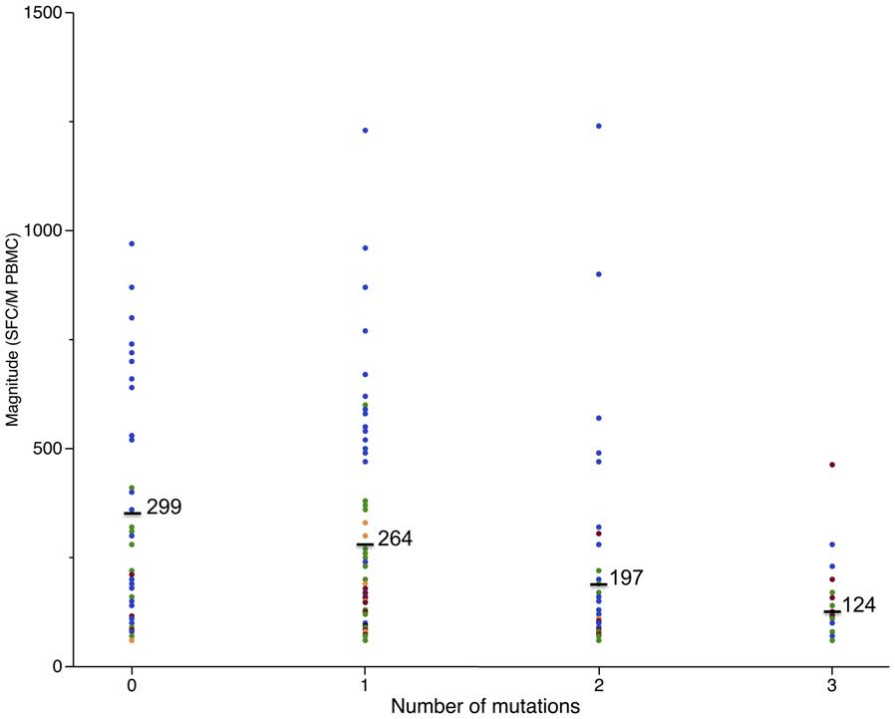
Magnitude of IFN-γ ELISpot responses toward peptides that do or do not match each individual's HIV-1 sequence. The graph shows the magnitude of ELISpot responses as a function of the number of mutations between the peptide tested and the individual's autologous HIV-1 consensus sequence. This analysis was performed for the five individuals in our cohort from whom HIV-1 sequences were available.

We compared the sequence of the peptides recognized by ELISpot to *nef* gene sequences from the infected individual, which were available for five individuals. When the subject's sequence matched the peptide sequence, the ELISpot response had a higher magnitude than when there was a mismatch between the peptide sequences and the individual's virus (Figure 5). There was a trend toward a decrease in the magnitude of responses with an increase of peptide/virus mismatches. The average magnitude of ELISpot responses was not significantly different if peptides exactly matched or had one mutation with the individual's sequences (p = 0.413), whereas the magnitude of the response was significantly lower when there were two (p = 0.002) or three (p = 0.039) mismatches.

### Lack of relationship between HIV-1-B Nef sequence diversity and CTL targeting

We next examined the relationship between the ELISpot recognition and the database variability of the HIV-1 segments that were recognized. We calculated the average Shannon Entropy for each 10-AA segment over the Nef protein based on an alignment of 514 known, independent sequences. The HIV-1 segments that were recognized were more conserved (i.e., with lower Shannon Entropy) than those that were never recognized (Figure S4; p<0.0001). Since Shannon Entropy is a measure of HIV-1 variability for each segment of a protein alignment, it assigns an identical entropy value for every 10-mer mapped to the same segment of HIV, i.e., it does not discriminate all the specific 10-mer-variants that have the same HXB2 coordinates.

**Figure 6:**
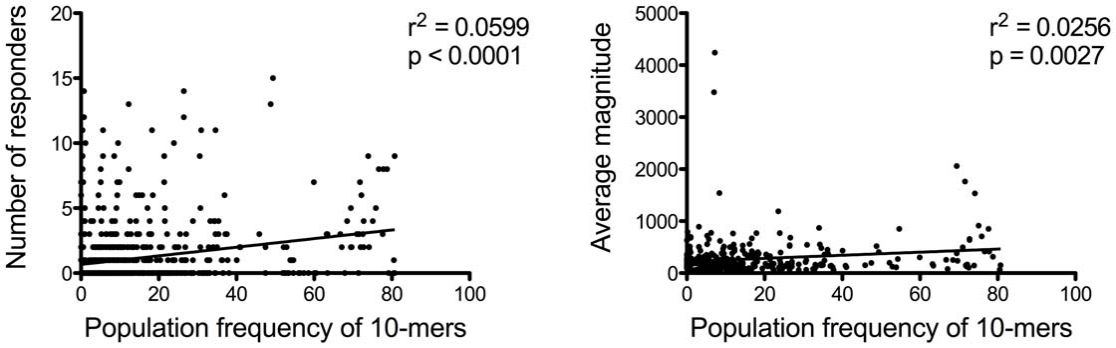
Population frequency of HIV-1-B Nef peptides and IFN-γ ELISpot responses. The frequency of each unique 10-mer in a dataset of 514 independent Nef HIV-1 subtype B sequences is shown. Peptide reactivity data generated by IFN-γ ELISpot assays done on 26 HIV-1 infected individuals were used to analyze the relationship between the population frequency of a peptide and either its frequency of recognition in the cohort (7A – left panel) or the magnitude of the IFN-γ ELISpot responses γ ELISpot response it elicited (7B – right panel).

Thus, we chose an alternative way to evaluate the impact of HIV-1diversity on ELISpot reactivity using a peptide-specific metric. Based on 514 independent Nef HIV-1 subtype B sequences, we calculated the population frequency of each unique peptide derived from this dataset, i.e., the percentage of sequences with the specific 10-mer. We found no relationship between the population frequency of a 10-mer and CTL targeting (Figure 6). There was no relationship between the frequency of a 10-mer in the population and its frequency of recognition among individuals: r^2^ = 0.0599 (p<0.0001; Spearman's correlation coefficient ρ was 0.2349, p<0.0001) (Figure 6A). Likewise, there was no relationship between the frequency of a 10-mer in the population and the magnitude of the ELISpot responses: r^2^ = 0.0256 (p = 0.0027; Spearman's correlation coefficient ρ = 0.1570, p = 0.0032) (Figure 6B). These results were confirmed when using a more recent and larger dataset of 1184 independent HIV-1 subtype B sequences (curated from sequences available at the HIVDB in Dec. 2009) (data not shown). Regarding the breadth of the CTL response, peptides that were common in the population, i.e., consensus-like, were recognized by an important proportion of our cohort – with up to 9 individuals recognizing the same peptide.

### Cross-reactivity among CTL responses

**Figure 7:**
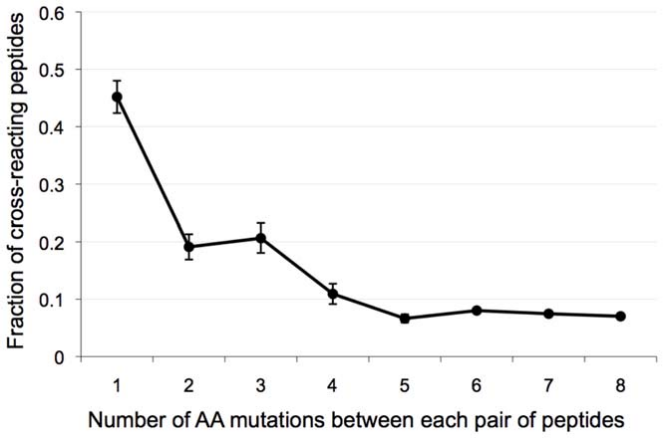
Cross-reactivity among HIV-1-B Nef peptides. The fraction of peptides that cross-react is represented as a function of the number of AA differences between the two peptides. For a pair of peptides (with the same HXB2 coordinates), the fraction of peptides that cross-react corresponds to the number of individuals who recognized both peptides divided by the number of individuals who recognized the more common one.

It is particularly striking that peptides that were rare in the population, i.e., found in less than 5% of circulating HIV-1 sequences, also elicited ELISpot responses in a number of individuals. For example, a 10-mer found in 0.78% of circulating sequences was recognized by 14 individuals (54% of our cohort). There was a striking example of a peptide (PGIRYP*I*TFG) found in 0.39% of database sequences that was nonetheless recognized by six individuals (average magnitude across the six individuals = 250 SFC/M), while the consensus epitope was not recognized (PGIRYPLTFG). To evaluate potential cross-reactivity between variant peptides, we examined pairs of peptides in which one peptide was found at least ten times more often among circulating sequences than the other peptide. The idea is that, in such situations, the reacting rare peptide is likely to be cross-reactive. Figure 7 shows the fraction of epitopes that putatively cross-react as a function of the number of AA differences between the two epitopes. We found that epitopes with a single AA substitution were likely to be recognized, and that recognition rates decreased as the number of AA changes increased. Furthermore, we found that the peptides are more likely to cross react if the AA changes were conservative (p = 0.03, 0.0044, 0.14 for 8, 9, and 10 mers, respectively).

## Discussion

Using a variation-encompassing library of 944 10-AA-long peptides that recapitulated much of the diversity found among circulating HIV-1 subtype B Nef peptides, we identified IFN-γ expressing responses against 350 peptides, including all but one of the known optimal epitopes in Nef, and revealed many novel epitope specificities after testing a set of only 26 HIV-1 infected individuals. The peptide library consisted of consensus 10-mers overlapping by 9 AA spanning the entire Nef protein and multiple variants covering the range of Nef diversity found in HIV-1-B infected individuals – with up to 8 peptidic variants for a consensus 10-mer. Our results suggest that variability-inclusive vaccine antigens, such as mosaic or COT+, can expand the breadth and depth of CTL responses, as shown recently in macaques [15], [16].

We demonstrated recognition of 297 Nef peptides in addition to the 53 (optimally-defined or not) epitopes that had previously been recorded in the HIVDB – underlining that our understanding of the determinants of epitope recognition is fairly limited. It is particularly significant that this is found for the oft-targeted Nef protein, suggesting that our knowledge of epitopes in more variable proteins such as Env is probably even more limited, as previously indicated [21]. The 26 individuals in this cohort targeted a median of 7 discrete epitopic regions in Nef (up to 32 for one individual), illustrating the common targeting of Nef peptides in chronically-infected individuals as previously reported [17]. The cross-sectional nature of our cohort and the availability of HIV-1 sequence data for only five individuals did not allow us to make inferences on the biological effectiveness of the responses we observed; indeed, we did not see any relationship between the viral loads of the individuals and the number of CTL responses they mounted or their magnitude. So far, CTL responses to Nef have not been associated with control of viral replication [11], rather they have been associated to higher viral loads compared to responses to Gag which have been associated to lower viremia [10], [11].

Overall, reactive peptides were more likely to correspond to more conserved portions of the HIV-1-B Nef protein - the 350 peptides that elicited ELISpot responses corresponded to viral regions of significantly lower Shannon entropy than the HIV-1 regions encompassing the 594 peptides that were not recognized (p<0.0001). Although many CTL responses were detected towards segments of lower Shannon entropy, highly variable segments were also immunodominant targets – in such cases only certain variants of a 10-AA-long viral segment could be recognized. Therefore, we used a measure of diversity that was specific for each 10-mer variant corresponding to the frequency of occurrence of each peptide among circulating HIV-1-B sequences based on a dataset of 514 recorded Nef sequences obtained from HIV-1-B infected individuals (using only independent sequences). We found no relationship between the frequency of a peptide and either the frequency of recognition in our cohort or the magnitude of the responses elicited.

We found that using a variability-enhanced peptide set increased the breadth and depth of CTL responses, suggesting that variability-inclusive vaccine strategies could elicit broader recognition of epitopes. Indeed, use of mosaic antigens was recently shown to enhance cellular immune responses in vaccinated monkeys [15], [16]. In variability-enhanced antigens, inclusion of common variants is favored and rare peptides are specifically excluded from the vaccine insert since these approaches were developed to compress HIV-1 variability in a vaccine insert of practical size (e.g., 2 to 4 gene lengths). The hypothesis is that common variants would be more likely to be found in infecting strains. We tested the recognition of all the peptides that would be included in 3 vaccine inserts and found that maximizing coverage in the vaccine insert using the COT+ method was useful to increase the number of peptides recognized. However, the mechanism behind the enhanced breadth appears different from a linear increase in recognition of epitopes due to increased-coverage of the viral diversity found among circulating HIV-1-B sequences. While a consensus peptide was generally the most likely to be recognized, unique or very rare peptides were also recognized. Of the 203 10-mer segments spanning Nef, 91 were recognized using consensus peptides and 46 additional segments were recognized using variants only (i.e., when the consensus was negative). We observed diminishing returns in epitope recognition as additional levels of variation (stratified by their population frequency) were tested. Interestingly, in the recent mosaic vaccine studies in monkeys, Santra et al. [16], using quadrivalent antigens, reported a weaker enhancement of T cell responses than did Barouch et al. [15] (using bivalent antigens). This difference may be associated in part with a decreasing effectiveness with a quadrivalent antigen compared to a bivalent one.

Unique viral peptides correspond to private mutations and represent a significant and growing proportion of the peptides found among circulating sequences, due to the extensive and continuously expanding diversity of HIV-1. We found that 61 peptides found in less than 1% of circulating sequences were recognized by at least one individual in our cohort, and 26 of these rare peptides were recognized by three or more individuals (and up to 14 individuals). For example, the consensus epitope (PGIRYPLTFG) was not recognized while its variant PGIRYP*I*TFG (found in 0.39% of database sequences) was recognized by six individuals. In this instance, it might be preferable to switch the consensus peptide for the L7I variant in a vaccine insert or peptide set in order to augment epitope recognition. On an individual basis, it was striking that most single mutations led to only modest and non-significant decreases in the amplitude of the T cell response. We showed that ELISpot responses elicited by these unique/rare peptides may have been due to cross-recognition with common variants: variants one AA away from the consensus peptides were the most likely to react, and this was particularly true if the AA substitution was conservative. Our data indicates that epitope binding to T cell receptors is promiscuous and conforms to the model proposed by McKinney and colleagues [22], which demonstrated that multiple AA changes engineered in epitopes still permitted CTL recognition in the context HLA-A*0201 and HLA-A*1101. The breadth of recognition of peptide variants is likely due in part to the promiscuity in TCR binding [23]; our results showed no significant difference in the magnitude of responses between variants, yet we could not draw conclusions as to whether the functional profiles of the responses differed.

It remains to be understood what role is played by cross-recognition of peptides in the control of viral replication. Knowing HIV-1's propensity to mutate, it is likely that several variants are generated under CTL pressure and a number of cross-reactive responses may be remainders of immune responses against initial or previous viral variants – it is crucial to determine under which conditions those variants cross-react and whether this has an impact on the efficacy of the anti-viral CTL response. If a multiplicity of peptides still induced substantial CTL responses without significantly compromising viral fitness, there may be a high genetic barrier to abolish CTL recognition. Hence protection by such an epitope might be explained by the complex patterns of mutations that are necessary for efficient escape.

Whether cross-reactivity has an effect on the CTL's ability to control viral replication is an open question that has important implications for vaccine design. If cross-reactivity can broaden the CTL response elicited by a vaccine and also has a positive impact on the control of viremia, intrinsic cross-reactive specificities of HIV-1 should be harnessed to develop a potentially more immunogenic vaccine candidate as a means to confer broadly protective immunity against multiple strains. However, the lack of association between the population frequency of an HIV-1 peptide and its recognition by individuals in the cohort reported here also suggests that an unrealistically large vaccine antigen size may be required to protect against the universe of viral strains capable of establishing an infection. In addition, since a number of responses were due to cross-reactivity between rare and frequent peptides, we surmised that a number of these very rare peptides, which are unlikely to have been found in the viruses from our infected subjects, may not induce efficacious anti-viral responses, but rather represent decoy responses. These results led us to discard our variability-inclusive COT+ vaccine strategy [13] in favor of a ‘Conserved Elements’ (CE) vaccine design [24]. CE vaccines seek to focus responses on the most conserved segments of HIV-1 in order both to elicit CTL responses considered obligatory for viral control and to avoid CTL responses toward variant peptides that allow escape without hindering viral function and that may act as immunological decoys with no clear efficacy in terms of viral control.

## Supporting Information

Figure S1 **Frequency of 3,831 10-mer peptides in a Nef protein dataset of 514 sequences.** Five hundred and fourteen Nef sequences were dissected into overlapping 10-mers. The majority of the 19,800 10-mer peptides were found only once (n = 13,574 peptides) or twice (n = 2,455 peptides) in the dataset and are not shown in the graph. The graph represents the 3,831 10-mer peptides that were found at least twice in the dataset and the number of occurrences of each peptide is figured.
(TIFF)

Figure S2 **Distribution of 944 10-mers along the Nef protein.** Gray bars represent the numbers of 10-mer peptides starting at each position along Nef. Values for each 10-mer are represented using their corresponding start position based on HXB2 coordinates. The black line corresponds to the average Shannon Entropy values calculated over overlapping 10-AA segments covering the Nef protein, based on an alignment of 514 independent sequences from HIV-1 subtype B.
(TIFF)

Figure S3 **Magnitude of IFN-γ ELISpot responses toward consensus and variant peptides.**
(TIFF)

Figure S4 **HIV-1-B Nef variability and IFN-γ ELISpot recognition.** Average Shannon Entropy scores were calculated for each 10-mer using an alignment of 514 independent sequences from HIV-1 subtype B. Peptide-specific Shannon Entropy values were compared based on their recognition in IFN-γ ELISpot assays done on 26 HIV-1 infected individuals.
(TIFF)

Table S1 **IFN-γ ELISpot responses detected against 944 Nef peptides (10-mer) in 26 HIV-infected individuals.**
(XLS)
