# Supplementary figures and images for: Increased Breadth and Depth of Cytotoxic T Lymphocytes Responses against HIV-1-B Nef by Inclusion of Epitope Variant Sequences
Source: PLoS One. 2011 Mar 28;6(3):e17969. doi: 10.1371/journal.pone.0017969 (PMC3065451; doi:10.1371/journal.pone.0017969)

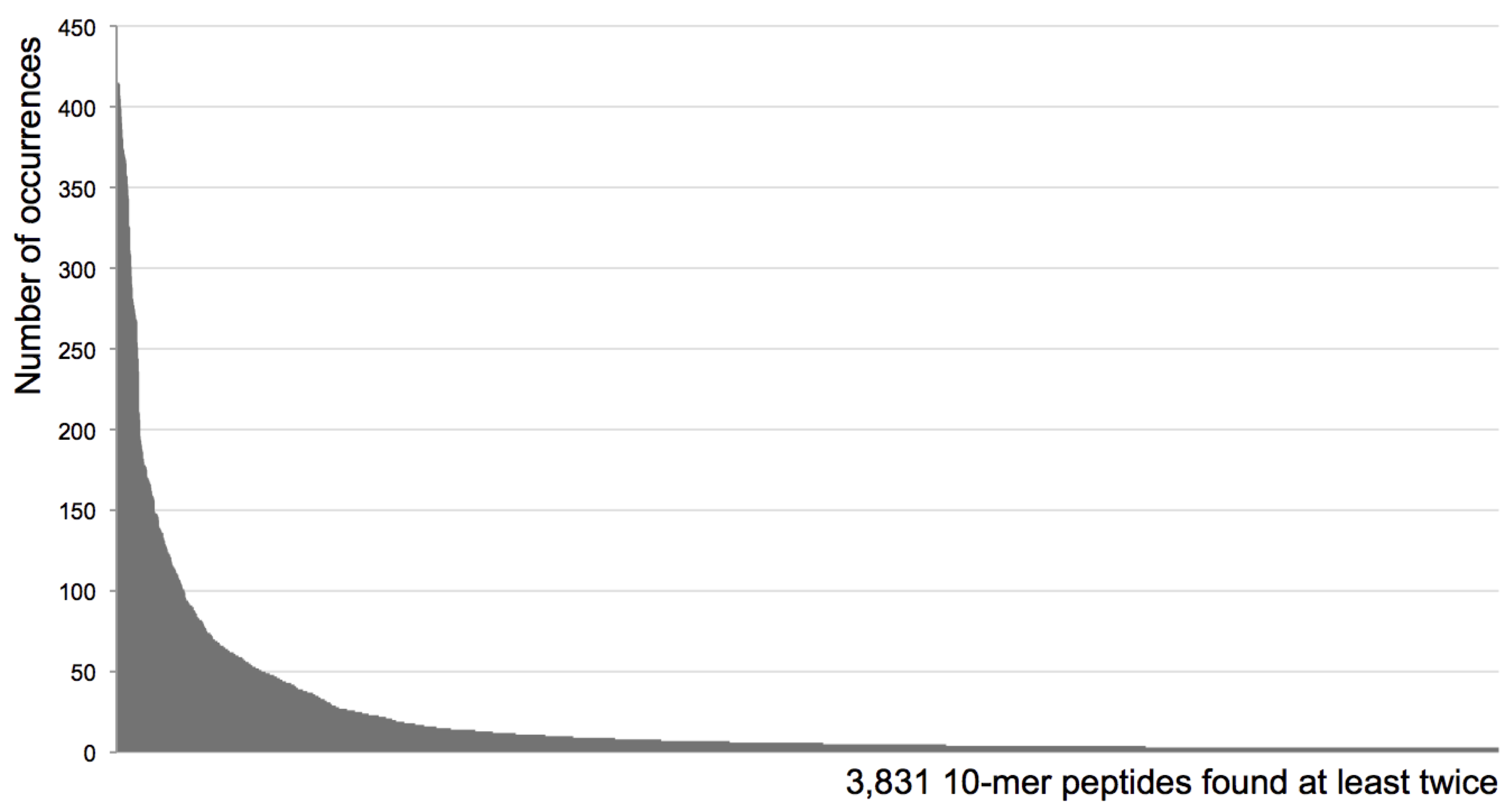

Supplement: Figure S1 — Frequency of 3,831 10-mer peptides in a Nef protein dataset of 514 sequences. Five hundred and fourteen Nef sequences were dissected into overlapping 10-mers. The majority of the 19,800 10-mer peptides were found only once (n = 13,574 peptides) or twice (n = 2,455 peptides) in the dataset and are not shown in the graph. The graph represents the 3,831 10-mer peptides that were found at least twice in the dataset and the number of occurrences of each peptide is figured. (TIFF) [file pone.0017969.s001.tif]

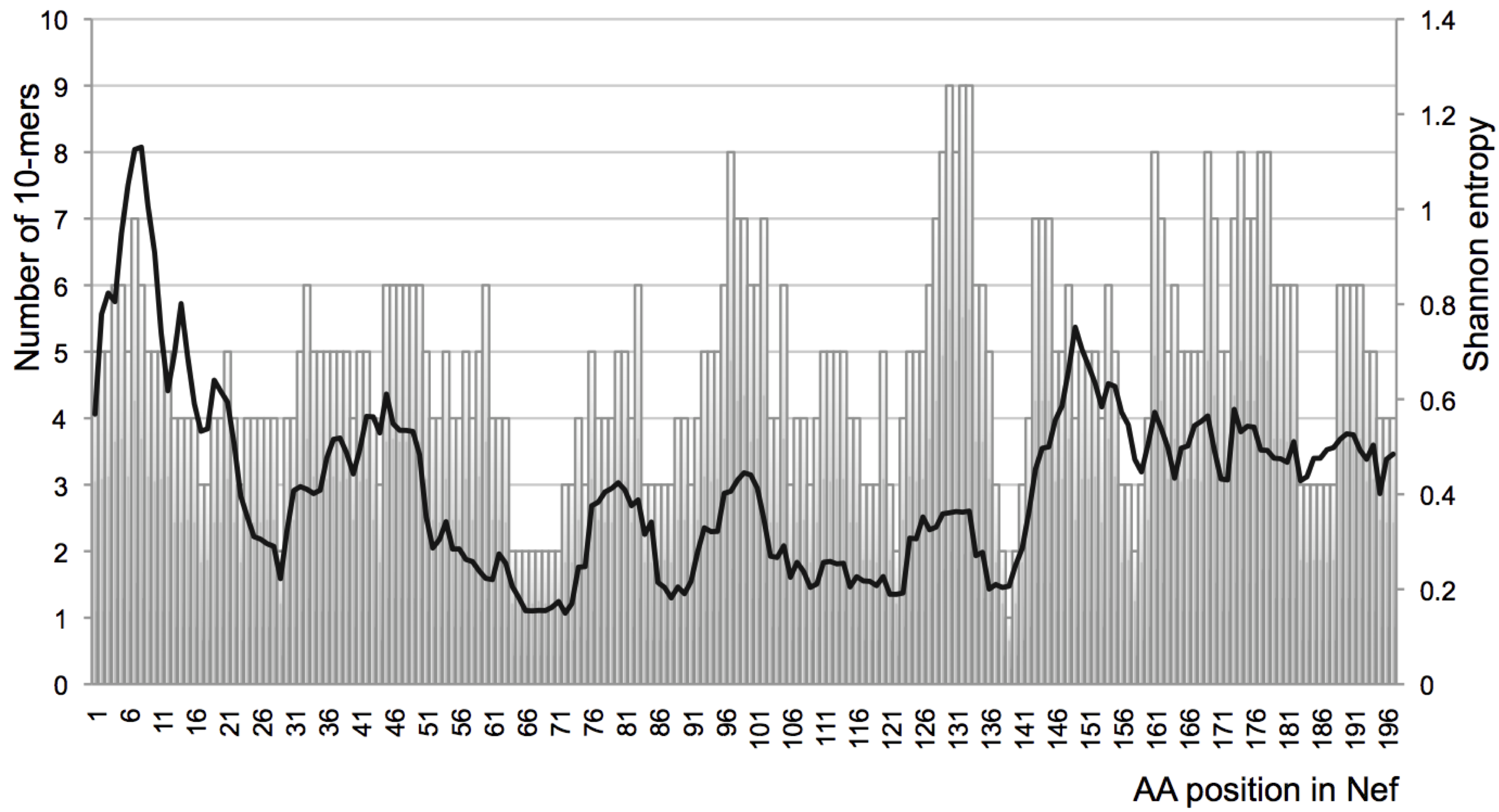

Supplement: Figure S2 — Distribution of 944 10-mers along the Nef protein. Gray bars represent the numbers of 10-mer peptides starting at each position along Nef. Values for each 10-mer are represented using their corresponding start position based on HXB2 coordinates. The black line corresponds to the average Shannon Entropy values calculated over overlapping 10-AA segments covering the Nef protein, based on an alignment of 514 independent sequences from HIV-1 subtype B. (TIFF) [file pone.0017969.s002.tif]

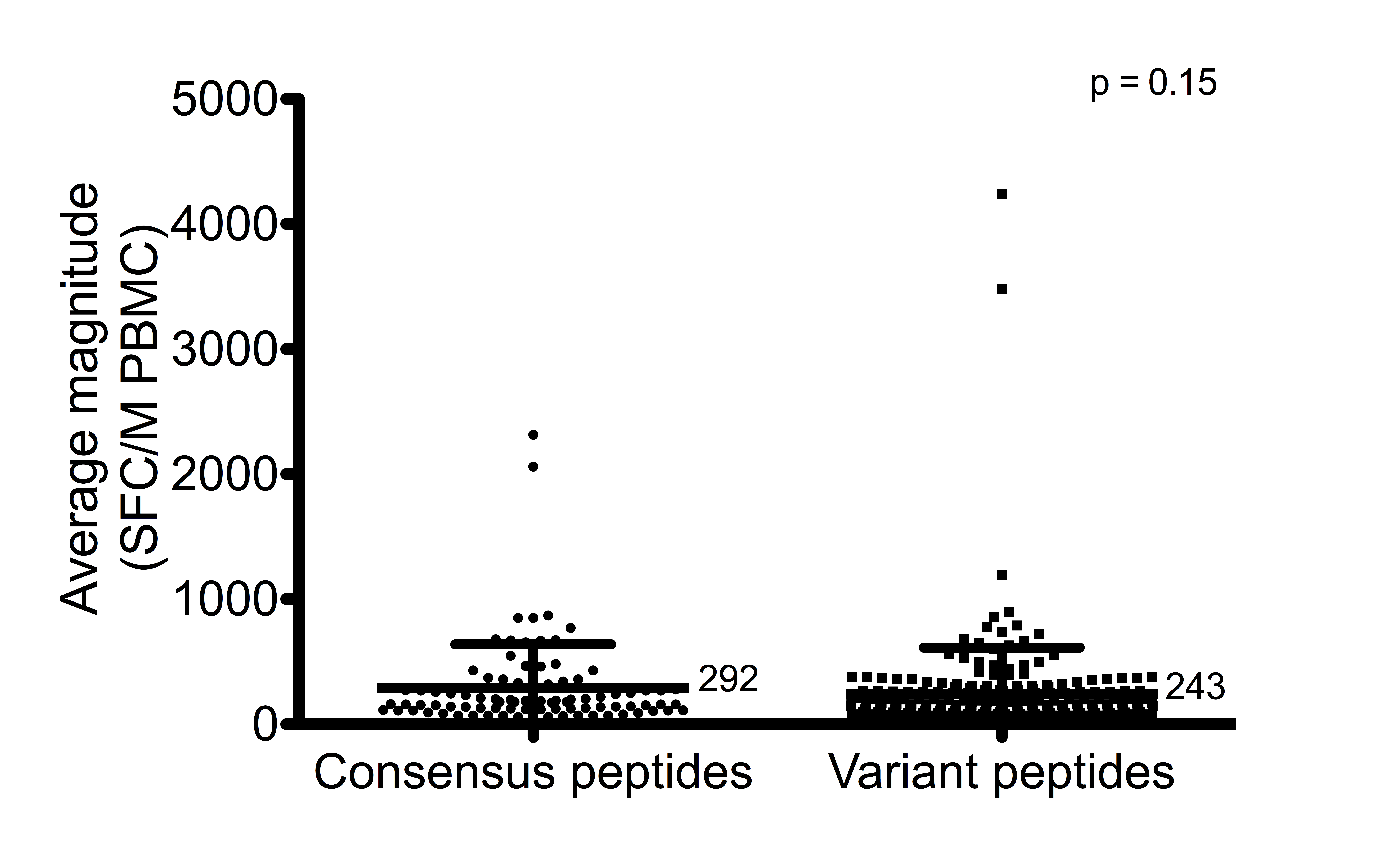

Supplement: Figure S3 — Magnitude of IFN-γ ELISpot responses toward consensus and variant peptides. (TIFF) [file pone.0017969.s003.tif]

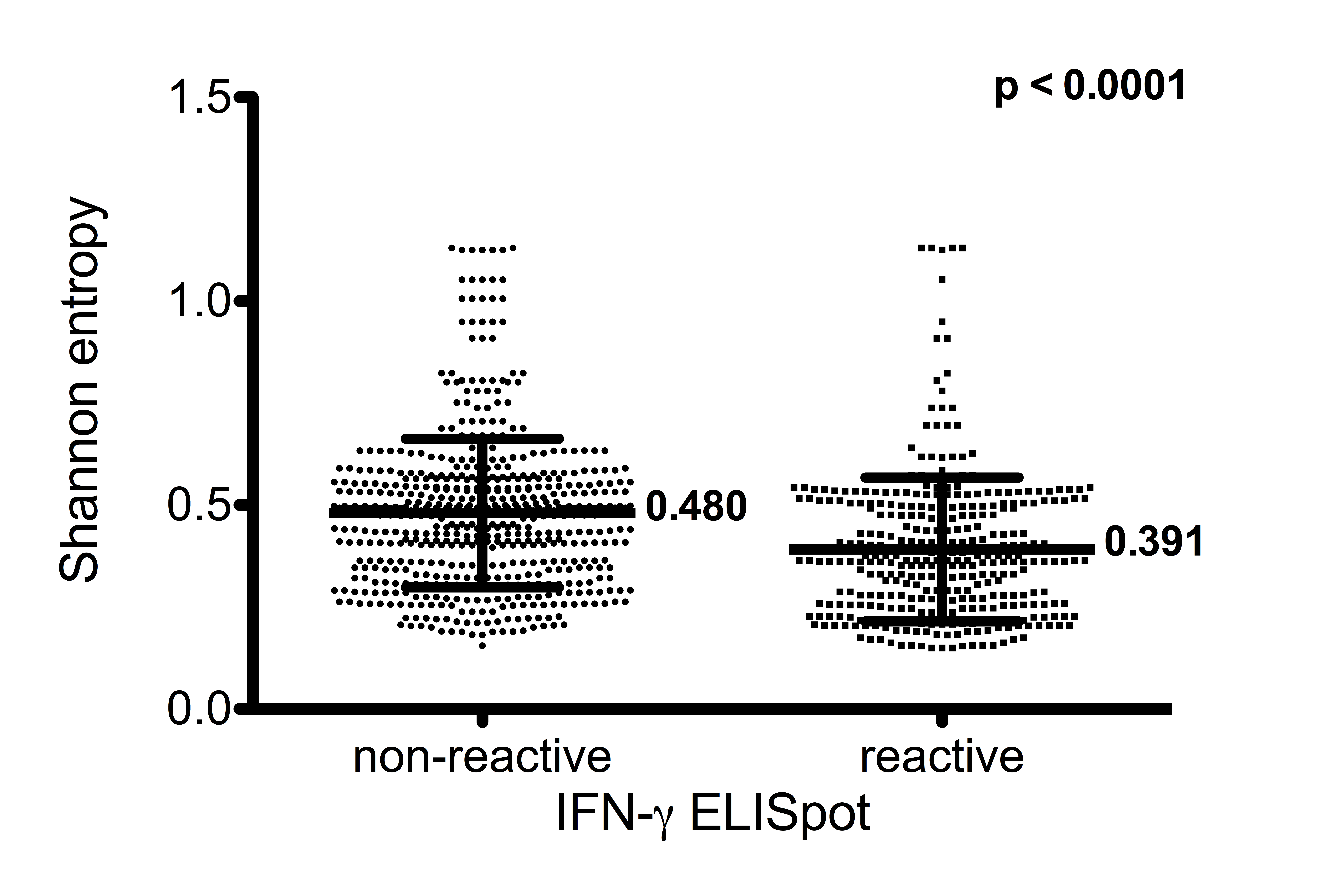

Supplement: Figure S4 — HIV-1-B Nef variability and IFN-γ ELISpot recognition. Average Shannon Entropy scores were calculated for each 10-mer using an alignment of 514 independent sequences from HIV-1 subtype B. Peptide-specific Shannon Entropy values were compared based on their recognition in IFN-γ ELISpot assays done on 26 HIV-1 infected individuals. (TIFF) [file pone.0017969.s004.tif]
